# A Novel Form of Compensation in the Tg2576 Amyloid Mouse Model of Alzheimer’s Disease

**DOI:** 10.3389/fncel.2016.00152

**Published:** 2016-06-16

**Authors:** Attila Somogyi, Zoltán Katonai, Alán Alpár, Ervin Wolf

**Affiliations:** ^1^Department of Anatomy, Histology and Embryology, Faculty of Medicine, University of DebrecenDebrecen, Hungary; ^2^Kenézy Gyula Hospital Ltd., Department of Emergency MedicineDebrecen, Hungary; ^3^MTA-SE NAP B Research Group of Experimental Neuroanatomy and Developmental Biology, Hungarian Academy of SciencesBudapest, Hungary; ^4^Department of Anatomy, Semmelweis UniversityBudapest, Hungary

**Keywords:** Alzheimer’s disease, human amyloid precursor protein, computer simulations, mouse somatosensory cortex, compensation, electrotonic analysis, conservation of dendritic signaling, synaptic integration

## Abstract

One century after its first description, pathology of Alzheimer’s disease (AD) is still poorly understood. Amyloid-related dendritic atrophy and membrane alterations of susceptible brain neurons in AD, and in animal models of AD are widely recognized. However, little effort has been made to study the potential effects of combined morphological and membrane alterations on signal transfer and synaptic integration in neurons that build up affected neural networks in AD. In this study spatial reconstructions and electrophysiological measurements of layer II/III pyramidal neurons of the somatosensory cortex from wild-type (WT) and transgenic (TG) human amyloid precursor protein (hAPP) overexpressing Tg2576 mice were used to build faithful segmental cable models of these neurons. Local synaptic activities were simulated in various points of the dendritic arbors and properties of subthreshold dendritic impulse propagation and predictors of synaptic input pattern recognition ability were quantified and compared in modeled WT and TG neurons. Despite the widespread dendritic degeneration and membrane alterations in mutant mouse neurons, surprisingly little, or no change was detected in steady-state and 50 Hz sinusoidal voltage transfers, current transfers, and local and propagation delays of PSPs traveling along dendrites of TG neurons. Synaptic input pattern recognition ability was also predicted to be unaltered in TG neurons in two different soma-dendritic membrane models investigated. Our simulations predict the way how subthreshold dendritic signaling and pattern recognition are preserved in TG neurons: amyloid-related membrane alterations compensate for the pathological effects that dendritic atrophy has on subthreshold dendritic signal transfer and integration in layer II/III somatosensory neurons of this hAPP mouse model for AD. Since neither propagation of single PSPs nor integration of multiple PSPs (pattern recognition) changes in TG neurons, we conclude that AD-related neuronal hyperexcitability cannot be accounted for by altered subthreshold dendritic signaling in these neurons but hyperexcitability is related to changes in active membrane properties and network connectivity.

## Introduction

Alzheimer’s disease (AD) is a pervasive neurological disorder, which is irreversible, progressive, and incurable at present. One characteristic hallmark of AD is the accumulation of amyloid-beta (Aβ) peptide in the form of insoluble extracellular senile plaques and soluble oligomers in susceptible areas of the brain. Although AD is probably a multifactorial illness, one of the most widely used working paradigms on the underlying mechanisms is the amyloid hypothesis. This hypothesis suggests that Aβ accumulation, primarily the soluble form of oligomers, is related to neurodegeneration, disrupts synaptic transmission and neural networks, and eventually leads to dementia (Hardy and Allsop, 1991; Hardy and Higgins, 1992; Hardy and Selkoe, 2002; Kung, 2012). Indeed, the level of soluble Aβ correlates with cognitive decline in AD (Fukumoto et al., 2010).

Neurons in layers II/III of primary somatosensory cortex represent one of the most severely affected neuron populations in AD (Hof et al., 1990; Hampel et al., 1998; Bussiere et al., 2003; Hof and Morrison, 2004). Changes in size of the corpus callosum and, more recently, its shape alterations have been correlated with the severity of AD (Zhu et al., 2012; Ardekani et al., 2014; Bachman et al., 2014). The function of callosal neurons in influencing cortical activity is not yet fully understood but their stimulation results in a strong net increase in contralateral excitation (Cisse et al., 2003; Karayannis et al., 2007; Petreanu et al., 2007; Ramos et al., 2008). Significance of callosal fibers in cortical excitation is highlighted by the observations that epileptiform activity may be evoked by repetitive electrical stimulation of callosal fibers and may be eliminated by callosal transection (Reeves and Roberts, 1986; Cisse et al., 2003; Ramos et al., 2008). Humans aﬄicted with AD are more prone to seizures than individuals in the control group (Amatniek et al., 2006; Lozsadi and Larner, 2006; Scarmeas et al., 2009). Furthermore, as activity of callosal neurons is involved in increasing the level of general excitation, cortical hyperexcitability in AD may partly be related to altered excitability of callosal neurons.

Various transgenic animal models have been developed to study the effects of Aβ accumulations at molecular, synaptic, single neuronal and network levels. Although, no animal model can reproduce the entire constellation of human symptoms of AD, these transgenic animals have contributed to our understanding of AD pathology (Ashe and Zahs, 2010; Luebke et al., 2010; Kitazawa et al., 2012). One of the most studied animal model is the Tg2576 transgenic (TG) mouse (Hsiao et al., 1996), which overexpresses the human amyloid precursor protein (hAPP) with the Swedish double mutation leading to progressive formation of soluble Aβ peptides and fibrillar amyloid plaques in brain tissue. This mouse shows Aβ-related neurodegenerative changes throughout its cortex and hippocampus. Neurites change their trajectory and diameter, lose spines and become dystrophic (Spires and Hyman, 2004; Tsai et al., 2004; Spires et al., 2005). These morphological alterations are accompanied by synaptic loss along with disturbance of the cortical network (Alpar et al., 2007) and the animal develops age-related cognitive impairment (Hsiao et al., 1996; Kishimoto et al., 2013). Layer II/III somatosensory neurons with callosal projections have also been found to show severe morphological alterations in APP mutant Tg2576 mice. The alterations included an overall drop in spine densities over the basal and apical dendrites, distorted total dendritic length and complexity of branching pattern and changes in dendritic diameters (Alpar et al., 2006; Rocher et al., 2008). On the other hand, passive membrane properties (resting membrane potential, input resistance, membrane time constant) and action potential firing properties (threshold, rise time, and amplitude) of the transgenic and age-matched control neurons remained the same as measured on the soma (Rocher et al., 2008). The pathological changes in dendritic morphology combined with the identical properties of action potential generation suggest the need for more detailed subcellular analysis to explore the possible functional consequences of morphological alterations in these AD-related neurons.

Therefore, we have studied whether the morphological alterations observed in these vulnerable neocortical neurons of Tg2576 mice are accompanied by changes of dendritic impulse propagation. This question is intriguing since attenuations and conduction delays of signals during their propagation along the dendrites determine properties of synaptic integration and affect firing properties of neurons. To address this potential process, we have compared the subthreshold dendritic impulse propagation and recognition of synaptic input patterns in layer II/III pyramidal neurons of the somatosensory cortex (S1) of wild-type (WT) and age-matched hAPP overexpressing Tg2576 mice by using compartmental cable models based on highly accurate spatial neuron reconstructions.

## Materials and Methods

### Ethics Statement

This study did not involve any human or animal subjects.

### Neuron Samples

Layer II/III commissural pyramidal neurons (*n* = 58) of the primary somatosensory cortex from three 11 months-old WT and three age-matched male Tg2576 mice were used for this study. These neurons were identical with those which have been studied morphologically by Alpar et al. (2006). Details on the tissue preparations, labeling procedures, and three-dimensional reconstruction of neurons have been described in the earlier paper (Alpar et al., 2006).

In brief, animals were deeply anesthetized and the parietal bone was partially removed, and biotinylated dextran amine (1 μl, 20%, BDA, Molecular Probes, Inc.) was delivered to the corpus callosum to label contralateral commissural neurons retrogradely. One week after BDA injection, anesthetized mice were transcardially perfused, brains were post-fixed in the same fixative and coronal sections were made in cryostat. After washing, sections were reacted with avidin-biotin-peroxidase complex (1:100 in TBS, VECTASTAIN Elite ABC kit, Vector Laboratories, Inc.) for 2 h and then visualized by 3,3′-diamino-benzidine (DAB, 0.025%, Sigma, St. Louis, MO, USA) and intensified with nickel-ammonium sulfate (0.05%, Merck, Darmstadt, Germany) in presence of hydrogen peroxide (0.001%), diluted in TBS. Following mounting, dehydration and covering, the neurons were traced by Neurolucida (Microbrightfield, Inc.) with an 100X immersions lens.

### Compartmentalisation of Neurons

The NEURON simulation tool (Hines and Carnevale, 1997, 2001) and the Neurolucida data files with the traced morphology of dendrites were used to create morphologically faithful segmental cable models of pyramidal cells from the WT and transgenic mice. All simulations were run in NEURON ver. 7.1-7.3 with a default integration time step of 0.025 ms. The model neurons consisted of a single soma compartment with 452 μm^2^ surface area (Alpar et al., 2006) and 28–101 dendritic compartments depending on the complexity of arborisation pattern and size of dendrites of individual neurons.

### Modeling Spines

Dendritic spines were assumed to be identical with 1.5 μm^2^ surface area (Larkman et al., 1992; Middei et al., 2008) and with linear densities of 1.5 and 2.0 spines/μm for the TG and WT neurons respectively (Rocher et al., 2008). Spines were modeled by proportionally increasing the specific membrane capacitance and conductance of dendritic compartments to account for electrical effects caused by the increase in dendritic surface area due to dendritic spines (Jaslove, 1992) without actually modifying the length and diameter of cylindrical compartments in our computational models. In the first step of this procedure the relative increase in total surface area (*q*) caused by spines was calculated for each dendritic compartment according to the formula q = (A_s_ + A_d_)/A_d_, where *A_s_* is the summed surface area of spines received by the dendritic compartment and A_d_ is the surface area of the “smooth” cylindrical compartment without the spines. Then, the adjusted specific membrane capacitance ($C_{m}^{*}$) and conductance ($G_{m}^{*}$) of the compartment were assigned individually for each compartment as $C_{m}^{*}=C_{m}\cdot q$ and $G_{m}^{*}=G_{m}\cdot q$, where C_m_ and G_m_ are the capacitance and conductance for the unit membrane area of the neuron without spines. This is a common way to model the global effects of spines on dendritic impulse propagation in a passive cable model, which was suitable since our purpose was to simulate signal propagation along the dendrites and not into or out of spines.

### Membrane Models of Neurons

Detailed comparative knowledge on the distribution and kinetics of different membrane conductances over the soma-dendritic membrane is not yet available for layer II/III pyramidal neurons of the somatosensory cortex of WT and Tg2576 mice. Therefore, we restricted our study to passive membrane, but simulations were performed in three significantly different membrane models (leaky dendrite, uniform, leaky soma models) representing a wide repertoire of possible conductance distributions over soma-dendritic membranes. We considered three soma-dendritic membrane models throughout our study to check our major results against variations in the precise details of soma-dendritic conductance distribution. The uniform membrane model assumed equal specific membrane resistances (transmembrane resistance for the unit area of plasma membrane) over the soma and dendrites (*R_ms_ = R_md_*), which was varied individually in each WT and TG model neuron until the 130 MΩ electrophysiologically determined mean neuron resistance was matched (Rocher et al., 2008). In the other two types of models non-uniform soma-dendrite membranes were assumed and the somatic and dendritic resistances were different with a step-change at the junction between the somatic and dendritic compartments. In these non-uniform models specific membrane resistance of either the soma or the dendrites was taken as 50 kΩcm^2^, and resistance of the other compartment was fitted to get an input resistance of 130 MΩ. Therefore, these non-uniform membrane models either represent a leaky soma (*R_ms_ < R_md_*) or a leaky dendrite (*R_ms_> R_md_*) approximation. Neuronal membranes are not likely to change abruptly at the soma-dendritic border; rather they may have continuously changing electrical properties with the distance from the soma. However, when the electrical behavior of a model neuron with step-change in the specific membrane resistance at the soma-dendritic border was compared to another model neuron with continuously changing membrane resistance according to a sigmoid function, the two neuron models were proved to be practically identical (Fleshman et al., 1988; Segev et al., 1990). Thus, our simplified step-change approximation of the possible spatial non-uniformity in soma-dendritic membrane resistance is unlikely to alter our simulation results significantly.

Specific membrane capacitance (*C_m_*) was assumed to be uniform over the soma-dendritic surface and was set in all three membrane models by fitting the membrane time constant (*τ*) of the model neurons to the electrophysiologically measured mean membrane time constant values (17.0 ± 1.9 and 16.9 ± 1.7 ms in the WT and TG neurons respectively, Rocher et al., 2008) by changing *C_m_* in 0.5 μF/cm^2^ increments. Finally, the axial resistivity (*R_a_*) was 150 Ωcm (Trevelyan and Jack, 2002; Kabaso et al., 2009) in all of our simulations.

To check our compensatory hypothesis (see later) 29 hypothetical TG’ neurons were also set up in all three membrane models. Morphology of these TG’ neurons were identical with those of the reconstructed TG neurons. However, specific membrane resistances and capacitances of TG’ neurons were not identical with those of TG model neurons, but were set up to mimic the healthy levels of these membrane properties as determined for model WT neurons. Estimated specific membrane resistances of WT neurons were higher than in TG neurons due to amyloid-driven changes in mutant TG neurons. Therefore, to set up specific resistances in TG’ neurons, we upscaled TG membrane resistances. For these scaling factors we used the ratios of mean specific membrane resistances determined earlier for WT and TG model neurons. Three ratios (scaling factors) were determined, one for each membrane model. This way we calculated the ratio of mean WT and TG level *R_md_* for the leaky dendrite model, the ratio of the mean WT and TG level *R_m_* for the uniform model, and the ratio of the mean WT and TG level *R_ms_* for the leaky soma model. These ratios were 1.09; 1.28 and 1.31, for the leaky soma, uniform and leaky dendrite models respectively. *R_md_*, *R_m_*, and *R_ms_* resistance values for TG’ neurons were calculated by multiplying corresponding resistances of model TG neurons by the above scaling factors. Specific membrane capacitances of TG’ neurons were set to 1.5, 2.0, and 2.0 μF/cm^2^, as found in healthy, WT neurons, in case of the leaky soma, uniform, and leaky dendrite models respectively.

### Initiation of PSPs and Measures of Dendritic Signal Transfer

Local synaptic activities (PSPs) with different kinetics received by the dendrites were simulated by steady-state and sinusoidal (*f* = 50 Hz) current injections to dendritic points. The number of injection sites (synaptic loci) per dendritic compartment was dependent on the length and specific membrane resistance of dendrites. The distance between two neighboring injection sites was never farther than 37 μm and 0.2 space constant, resulting in 42–260 injection sites per neuron.

Somatopetal attenuations of PSPs between injection sites and the soma were quantified by the steady-state and sinusoidal (50 Hz) voltage transfers and current transfers. *Voltage transfer* was defined by the ratio of the amplitude of somatic membrane potential change and the amplitude of dendritic membrane potential change at the site of current injection when constant or sinusoidal current was injected. *Current transfer* was defined as the ratio of electrical charge reaching the soma divided by the total electrical charge injected at the dendritic point. Time delays associated with signal propagation between dendritic points and the soma along the dendrites were quantified by propagation delays measuring the time needed for the centroid of the voltage-time curve to reach the soma. Therefore, *propagation delays* measure how quickly a membrane potential change caused by activity of a synapse with a given dendritic location can spread to the soma. Time delays between synaptic current flow through the post-synaptic membrane and the developing local voltage response were quantified by *local delays* measuring the time delay between the centroids of the voltage-time and current-time curves at the site of current injection (Agmonsnir and Segev, 1993; Zador et al., 1995). The sum of propagation and local delays for a dendritic synapse measures the total time elapses between synaptic current flow at a working dendritic synapse and the development of somatic voltage response. These measures of PSP attenuations and delays were computed in the apical and basal dendrites separately to account for the differing morphological alterations in the two arbors found in transgenic animals (Alpar et al., 2006). Attenuations and delays of PSPs were graphed as the function of path distance between the site of PSP generation and the soma to reveal distance-dependence of these parameters. To estimate the weight of a given attenuation or delay among the many location-dependent values, the distributions of dendritic surface area as a function of PSP transfers/delays were also computed. These *comparison graphs*, showing distance-dependence and weights of dendritic signal transfers/delays were used to quantify differences between neuron classes and these graphs are presented in the Supplementary material.

Finally, to test if our major findings are independent of natural variations in size of neurons, distance-dependence of attenuations and delays as well as distributions of dendritic surface area in the function of transfers/delays were also analyzed over normalized scales. In these analyses over normalized scales path distances and attenuations/delays were measured as percentage of their maximum values in neurons. Summarized data on comparisons of different neurons over normalized scales are presented in the Supplementary material.

### Measuring Synaptic Input Pattern Recognition Capabilities in WT and TG Neurons

In a recent paper (de Sousa et al., 2015) ability of synaptic input pattern recognition was tested in a huge number of model neurons with different morphologies and with passive and active membrane properties. In their computational model a Hebbian learning rule (a kind of use-dependent synaptic facilitation) was applied: each dendritic compartment of the post-synaptic neuron received one synapse. A given pattern of synaptic inputs to the post-synaptic neuron was created by simultaneous activation of 10% of randomly chosen synapses. A number of different randomly generated synaptic input patterns were generated and strengths (conductances) of synapses were varied (at the beginning all synapses were equal). By the end of this “learning phase” each synaptic conductance was increased in proportion with the number of times the given synapse got activated during the learning phase (in the different synaptic input patterns). Then, output (EPSP amplitude or number of spikes) of the “trained” post-synaptic neuron, with these adjusted synaptic conductances, was measured in response to randomly chosen new, “novel” synaptic input patterns, where all synaptic conductances were at the original (non-varied) level. Finally, synaptic input pattern recognition capability was quantified by the ratio of somatic EPSP amplitudes (or by the ratio of the number of spikes produced) in the post-synaptic neuron in response to “learnt” and “novel” synaptic inputs to its passive (or active) dendrites. These ratios, therefore, measure the extent to which a “trained” neuron can discriminate between “learnt” and “novel” synaptic input patterns. The authors found that two simple metrics, the mean electrotonic distance of synapses and the within-cell variance of these electrotonic distances, correlated inversely with the pattern recognition capacity of neurons with both passive and active dendritic membranes (de Sousa et al., 2015).

Based on this very detailed study, capacity of WT and TG neurons to discriminate between “learnt” and “novel” dendritic synaptic input patterns was predicted and compared by using the mean electrotonic distance of synapses and the within-cell variance of these electrotonic distances in our neurons. Mean electrotonic distance of synapses was estimated by averaging electrotonic distances of the midpoints of dendritic segments from the soma in each WT and TG neuron individually according to the following formula: 1/n∑ ∏ _i_, where n is the number of dendritic segments in the neuron, and ∏ _i_ is the electrotonic distance of the midpoint of the *i^th^* dendritic segment from the soma.

### Statistical Analysis

For statistical analysis and plotting the figures the Microsoft Office (Microsoft Corp.), PAST (Hammer et al., 2001) and SPSS (SPSS, Inc., Chicago, IL, USA) software products were used. To compare means of attenuations and delays of PSPs the Mann–Whitney test was used. To compare the mean of data from simulations with the experimentally derived mean value the one-sample *t*-test was applied. Distributions of dendritic length and surface areas were compared by the Kolmogorov–Smirnov test. Significance level was chosen to be 0.05 in all statistical tests. In quantitative data arithmetic means are followed by standard errors of means (SEM) that were also used as error bars in the figures.

## Results

### Distribution of Dendritic Surface Area as a Function of Path Distance From the Soma Approximates the Distribution of Excitatory Synapses Received by Dendrites of WT and TG Pyramidal Neurons

**FIGURE 1:**
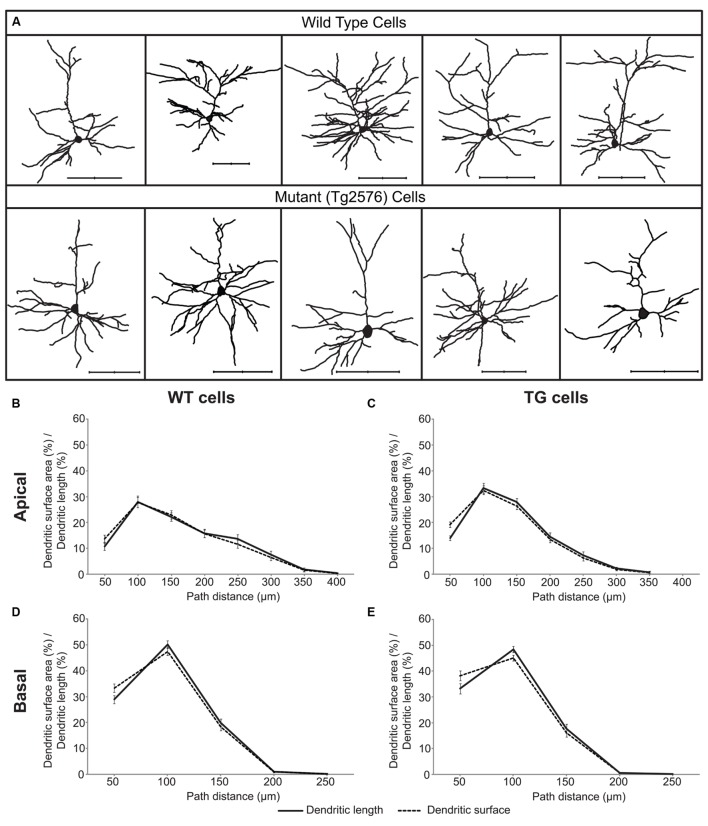
**A sample of layer II/III wild-type (WT) and transgenic (TG) neurons of Tg2576 mice (A) and distributions of their dendritic surface area (dashed lines) and dendritic length (solid lines) as a function of path distance from the soma in apical (B,C) and basal (D,E) dendrites.** Distributions of dendritic surface area and length were statistically identical (Kolmogorov–Smirnov test, *p* > 0.7) both in the apical and in the basal dendritic arbors of WT and TG neurons. Scale bars 100 μm.

We investigated if Aβ exposure leads to altered subthreshold dendritic impulse propagation and synaptic input pattern recognition in Tg2576 neocortical neurons or morphological degeneration may be compensated by accompanying Aβ-driven changes in membrane properties. For easy reference we used the very same WT and TG neurons (**Figure 1A** and **Table 1**) whose morphology have been studied and reported earlier (Alpar et al., 2006).

**Table 1:** Compiled quantitative morphological data showing some of the differences between wild-type (WT) and transgenic (TG) cortical neurons of Tg2576 mice.

Morphological parameter			WT	TG	*p*
Total	Dendritic length of apical dendrites^1^ [μm]		1083 ± 92	806 ± 60	*p* < 0.05
	Dendritic surface area including spines^2^ [μm^2^]	Basal	1448 ± 80	1148 ± 77	*p* < 0.005
		Apical	5189 ± 434	3242 ± 209	*p* < 0.001
Overall	Spine density^3^ [spines/μm]		2.0	1.5	
	Dendrite curvature ratio^4^		0.99 ± 0.01	0.96 ± 0.04	*p* < 0.05
Branching	Total number of oblique dendrites^1^		4.78 ± 0.33	3.27 ± 0.28	*p* < 0.05
	Total number of bifurcations in apical dendrites^1^		14.9 ± 1.3	10.0 ± 0.7	*p* < 0.05
Average diameter of apical dendrites by order^1^ [μm]	Shaft		0.96 ± 0.06	1.13 ± 0.05	*p* < 0.05
	OT1		0.57 ± 0.02	0.68 ± 0.04	*p* < 0.05
	OT2		0.45 ± 0.02	0.52 ± 0.03	ns
	OT3		0.41 ± 0.03	0.38 ± 0.03	ns
	OT4		0.36 ± 0.02	0.26 ± 0.02	*p* < 0.05
	OT5		0.32 ± 0.02	0.22 ± 0.02	*p* < 0.05
	T1		0.71 ± 0.03	0.98 ± 0.05	*p* < 0.05
	T2		0.57 ± 0.04	0.65 ± 0.03	ns
	T3		0.39 ± 0.05	0.41 ± 0.04	ns

*Data are from ^1^(Alpar et al., 2006), ^2^calculations by the authors, ^3^(Rocher et al., 2008), ^4^(Spires et al., 2005). OT: oblique dendrites, T: apical tuft.*

During our investigations one of our aims was to characterize features of dendritic signal propagation by estimating the percentage of dendritic synapses associated with a given range of somatopetal PSP transfers or delays. This allowed us to use percentages of synapses as natural weights for location-dependent properties of PSP propagation in a neuron. Here, we describe the morphological basis of this weighting.

In neocortical pyramidal neurons the vast majority (over 90%) of excitatory synapses are received by dendritic spines and usually one spine receives one synapse (Nimchinsky et al., 2002) and only 3.6% of spines lacks synapses in the mouse neocortex (Arellano et al., 2007). Thus, distribution of dendritic spines readily estimates the distribution of excitatory synapses. Linear spine density (number of spines/micrometer dendrite) is nearly constant in layer II/III pyramidal neurons of the somatosensory cortex in WT and TG mice (Rocher et al., 2008), indicating distribution of dendritic length to be a good estimator of spine and excitatory synapse distributions. We compared the distributions of dendritic surface area and dendritic length as a function of path distance from the soma in 29 WT and 29 TG neurons, and found that these distributions were not significantly different (**Figures 1B–E**) neither in WT nor in TG neurons. This result indicates that in these neurons, percentage of dendritic surface area is a good estimator for the percentage of the total number of excitatory synapses received over the respective dendritic surface.

Distribution of inhibitory synapses is more difficult to assess because of the lack of direct relationship between dendritic spines and inhibitory synapses, as these synapses are mostly received by dendritic shafts. However, the percentage of symmetrical to asymmetrical synapses was found to be nearly uniform on reconstructed dendritic shafts in the mouse primary somatosensory cortex, and the ratio of the total symmetric to asymmetric synapses was also constant (Hersch and White, 1982). These findings suggest a correlation between the number of inhibitory synapses and the surface area of dendritic segments in somatosensory cortex. A number of other studies have also presented evidence for a close relationship between the size of dendritic receptive surface and the number of synapses and spines (Colonnier, 1968; Feldman, 1975; Feldman and Dowd, 1975; Muller et al., 1984). Therefore, we used percentages of total dendritic surface area of neurons as estimators for the fractions of the total number of dendritic synapses that are able to generate PSPs with similar propagation properties.

### Building Passive Segmental Cable Models of WT and TG Neurons

**FIGURE 2:**
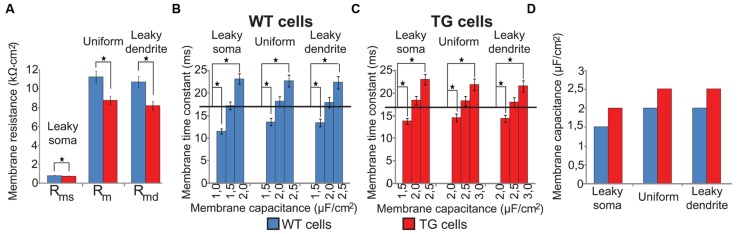
**Specific membrane resistances and capacitances of modeled (WT, blue bars) and transgenic (TG, red bars) neurons in three soma-dendrite membrane models. (A)** Specific membrane resistances were significantly smaller in TG than in WT neurons in all models. **(B,C)** Specific membrane capacitances of model WT and TG neurons were estimated by fitting simulated membrane time constants to their physiological values (black horizontal lines). **(D)** Specific membrane capacitance was bigger in TG than in WT neurons in all membrane models. Asterisks mark significantly different mean values (Mann–Whitney test, *p* < 0.05). Experimentally measured neuron resistances were 127.7 ± 9.0 MΩ in WT vs. 134.5 ± 9.2 MΩ in TG neurons and the difference was not significant (Rocher et al., 2008). In computer models of these neurons we used a common 130 ± 0.1 MΩ input resistance.

Specific membrane resistance and capacitance values were not directly available for WT and TG neurons but they were needed to create segmental cable models of WT and TG neurons. First, specific membrane resistances were estimated by injecting constant current to the soma of our morphologically detailed cable models of WT and TG neurons and by varying specific membrane resistances. Specific membrane resistances were varied until somatic DC input resistance, calculated from somatic voltage changes in response to current injection, became equal to the experimentally measured mean resistance value (Rocher et al., 2008). These fitted specific membrane resistances in TG neurons were significantly lower than in WT neurons (Mann–Whitney test, *p* < 0.003, **Figure 2A**) in all membrane models due to the changed morphology of TG neurons. This decreased membrane resistance of TG neurons is in agreement with the Aβ-induced membrane permeabilization and channel formations resulting in membrane conductance increases reported earlier in a number of biophysical studies (Arispe et al., 1993; Kayed et al., 2004; Sokolov et al., 2004; Valincius et al., 2008). In line with biophysics, observations using atomic force microscopy and high resolution transmission electron microscopy also showed bilayer surfaces with Aβ-linked disturbances and channel formations in artificial membranes (Green et al., 2004; Quist et al., 2005), as well as in neuronal membranes of both APP transgenic mice (Kokubo et al., 2009) and AD patients (Inoue, 2008).

Then, specific membrane capacitances (*C_m_*) were determined by varying *C_m_* until membrane time constants of model neurons matched the electrophysiologically determined neuronal membrane time constants of these neurons (Rocher et al., 2008). Membrane time constants were calculated from voltage responses of model neurons to depolarizing current steps. These fits of membrane time constants resulted in 1.5, 2.0, 2.0 and 2.0, 2.5, 2.5 μF/cm^2^ capacitances for the WT and TG neurons in case of the leaky soma, uniform, and leaky dendrite models respectively. None of the membrane time constants calculated with the above capacitances differed significantly from their respective electrophysiological values (17.0 and 16.9 ms in WT and TG neurons, one sample *t*-test, *p* > 0.1) but increasing or decreasing *C_m_* by 0.5 μF/cm^2^ resulted in *τ* values significantly different from the experimental ones (one sample *t*-test, *p* < 0.01, **Figures 2B,C**). Membrane capacitance was predicted to be higher in TG than in WT neurons by all of our computer models (**Figure 2D**) based on fitting physiological time constants. This is, again, in line with the soluble prefibrillar Aβ(1–42) oligomers-caused, geometrical thinning of bilayers and with the increase of membrane dielectric constant observed experimentally, both of which lead to an increase of membrane capacitance (Valincius et al., 2008).

### Computed Features of Dendritic Impulse Propagation and Their Significance

*Current transfers, steady-state and 50 Hz sinusoidal voltage transfers* were used to describe attenuations of PSPs between the various dendritic points and the soma. The rationale behind using these transfers was that activity of neurons (chances of action potential generation) could be related to the ability of dendrites to conduct electrical charge and to transfer voltage perturbations of dendritic membrane toward the soma (nearby axon hillock) during activity of synapses. Voltage transfers are frequency-dependent due to the membrane capacitance and, therefore, voltage transfers (attenuations) of PSPs with slow and fast time constants are different. This is why voltage transfers were studied at steady-state and at 50 Hz also. *Local delays* were computed to characterize time delays between the development of PSP and the synaptic current at the site of simulated dendritic synapse and *propagation delays* were applied to quantify time needed for the signal to reach the soma once PSP has been generated at a dendritic location (Agmonsnir and Segev, 1993; Zador et al., 1995). The sum of the local and propagation delays give the total delay between the somatic voltage change and the synaptic current flow in dendrites. These delays are important when multiple synapses are active simultaneously and temporal summation of overlapping PSPs determine somatic membrane potential changes. All transfers and delays were computed in three different soma-dendritic membrane models of each of the 29 WT and 29 TG neurons to check if our major conclusions are critically dependent on assumptions about the (unknown) distribution of membrane resistances over the soma-dendritic surface.

### Virtually Identical Subthreshold Dendritic Impulse Propagation in TG and WT Neurons

**FIGURE 3:**
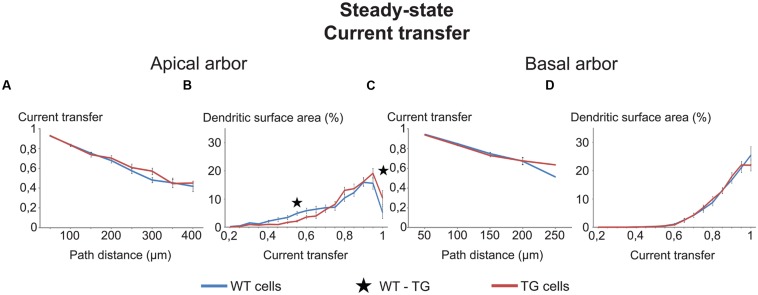
**Comparison graphs on currents transfers in WT and TG neurons.** Distance dependence of current transfers **(A,C)** and percentages of total dendritic surface area **(B,D)** with different rates of current transfers in uniform models of (WT, blue lines) and transgenic (TG, red lines) neurons. Black asterisk marks the only range where TG and WT neurons were different (Mann–Whitney test, *p* < 0.05). Note that some lines run very close to one another and some parts of lines remain hidden.

To compare dendritic signaling in WT and TG neurons transfer and delay properties of dendritic impulse propagation were computed for multiple dendritic locations in each neuron and two types of *comparison graphs* were created (see **Figure 3** as an example). In one type of comparison graphs dependence of transfers and delays on the distance of PSP generation site from the soma was graphed. In the other type of comparison graphs percentages of dendritic surface areas (∼percentage of synapses) associated with given transfer/delay values for the locally generated PSPs were graphed. Similarity of subthreshold dendritic signaling in WT and TG neurons was compared by percentages of ranges in comparison graphs, where statistically significant difference was found between mean values of transfers/delays of WT and TG neurons relative to the total number of ranges in graphs, where WT-TG statistical tests were performed. Therefore, 100% difference would mean that dendritic impulse propagation is entirely (in all ranges) different in WT and TG neurons, while 0% difference means no difference in a given feature of dendritic signaling.

The two types of comparison graphs were made for each transfer (current, steady-state and 50 Hz sinusoid voltage) and delay (local and propagation) we computed. We exemplify comparison graphs with the current transfer in the uniform model. These transfers were statistically identical both in the apical and basal dendrites of WT and TG neurons from all path distance regions (**Figures 3A,C**). This indicated equal efficiencies of synapses of TG and WT neurons in affecting the soma potential due to the identical efficiency of spread of electrical charge toward the soma and (nearby axon hillock) during synaptic current flows in dendrites (assuming other factors equal).

Parallel with this, some tendency of redistribution of dendritic surface area was observed in the function of current transfers in the apical, but not in the basal dendrites of TG neurons. This redistribution decreased the percentage of total dendritic surface area in TG neurons, where synapses had low (smaller than 0.75) transfers of synaptic current toward the soma (**Figures 3B,D**). At the same time, the redistribution increased the percentage of dendritic surface area where synapses had high current transfers (bigger than 0.75). Therefore, due to this redistribution, a bigger percentage of synapses could operate with high current transfers and a smaller percentage of synapses operated with low current transfers, resulting in a higher mean transfer rate of synaptic currents to the soma in TG than in WT neurons. This redistribution, if it was present in many transfer ranges, could be important under massive synaptic activities, involving many synapses with nearly homogeneous distribution of active synapses over the dendrites. In this case, assuming all other features identical, a massive redistribution of dendritic surface area could lead to excitability changes in TG neurons. Since the redistribution of dendritic surface area was not significant in most of the current transfer ranges (**Figures 3B,D**), these excitability changes are unlikely to be significant and cannot alter excitability of TG neurons. Taken together, neither the location-dependence of current transfers, nor the percentage of synapses with different transfers changed in TG neurons. Thus, excitability changes, observed experimentally in Tg2576 mouse neurons, must be attributed to other than current transfer changes in the AD-related animal.

**FIGURE 4:**
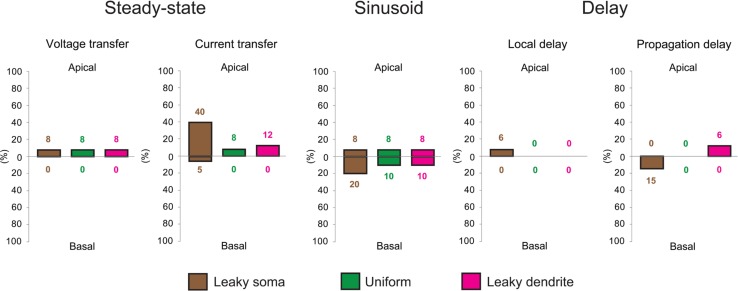
**Aβ-driven alterations in steady-state current- and voltage transfers, 50 Hz sinusoid voltage transfers and local- and propagation delays of PSPs in TG neurons relative to control, WT neurons.** Comparisons of dendritic impulse propagation were summarized in the leaky soma (brown), uniform (green) and leaky dendrite (magenta) membrane models. Size of bars illustrates the percentage differences between WT and TG neurons in certain features of dendritic impulse propagation. Upper and lower extensions of bars show the size of differences in properties of apical and basal arbors of WT and TG neurons. Data summarized here are based on comparison graphs shown in Supplementary Figures S1–S5.

Data based on all comparison graphs (not shown here), describing multiple features of dendritic signaling were summarized in (**Figure 4**). In general, all features of dendritic signaling were found to be very similar in TG and WT cells regardless of the membrane model assumed. The only notable exception, in which TG and WT neurons differed considerably, was the current transfer in the apical dendrites in leaky soma model. In this model the soma acted as a shunt and dendrites had higher specific resistivities resulting in the model with the highest sensitivity to dendritic morphology among our models. This explains the relatively bigger difference between signaling of the apical dendrites of WT and TG neurons, as morphology of apical dendrites is altered significantly in TG neurons. Independently of the membrane model, apical dendrites rather than the basal dendrites of TG neurons tended to differ more from their WT counterpart in their steady-state voltage-, and current transfers and local delays. This is in line with the more severe morphological changes in the apical dendrites, presumably affecting spread of PSPs more in the apical than in the basal dendritic arbor. The extent of differences in sinusoid 50 Hz voltage transfers between transgenic and non-transgenic neurons was similar in the basal and apical dendritic arbors (with the exception of leaky soma model). Sinusoid voltage transfers at 50 Hz differed more than steady-state voltage transfers in basal dendrites but not in the apical dendrites of WT and TG neurons. The frequency-dependent alterations of voltage transfers in TG neurons predicted that somatopetal transfer of slow, NMDA-dependent, and fast, AMPA-mediated, PSPs were affected to different degrees by amyloidosis. The imbalance between AMPA and NMDA-mediated synaptic systems in AD has also been described in a number of experimental studies (Di Lazzaro et al., 2003; Farlow, 2004; Revett et al., 2013).

In terms of the different soma-dendrite membrane models, overall transgenic dendritic signaling was altered the most in the leaky soma model, while alterations remained smaller in the uniform and leaky dendrite models, where percentage differences in PSP propagation properties never exceeded the 12% value (**Figure 4**). The model-independent observation of surprisingly limited alterations in TG dendritic signaling strongly supported that such conservation of subthreshold dendritic signaling was not dependent on the choice of soma-dendritic membrane models but represented a robust, biologically relevant phenomenon.

### Neuronal Membrane Changes Compensate for Pathological Subthreshold Dendritic Impulse Propagation Caused by Morphological Degeneration in Alzheimer-Related TG Neurons

The surprisingly similar dendritic signal propagation in TG and WT neurons, despite the severe morphological degeneration of TG cells, led us to the compensatory hypothesis: the amyloid-caused changes in biophysical characteristics of the neuronal membrane of layer II/III somatosensory TG neurons may act to compensate for parallel going morphological degradation and results in preservation of subthreshold dendritic signaling in neocortical neurons of the AD-related mutant animal. This proposed mechanism that tends to keep dendritic signaling unaltered could be vital for healthy synaptic integration, a process essential in neurons integrating signals from multiple afferents arriving from different sources.

The compensatory hypothesis was tested by comparing the number of ranges where dendritic signaling was different in WT vs. TG and WT vs. TG’ comparisons (see Materials and Methods for details on construction of hypothetical TG’ neurons with amyloid-related pathological morphology but with healthy membrane properties). The rationale behind this was that if our hypothesis is valid, then dendritic impulse propagation of TG’ neurons should, in tendency, differ more from that of WT cells than TG cells differed from WT cells in TG-WT comparisons. This is because of the lack of presumed compensatory membrane alterations in TG’ neurons, while this compensation could happen in TG neurons with amyloid-related changes in electrical properties of the soma-dendritic membrane. Such compensation by altered membrane properties may ensure TG neurons to preserve features of healthy dendritic PSP propagation found in WT cells, consequently less difference in dendritic signaling is expected in WT-TG than in WT-TG’ comparisons.

**FIGURE 5:**
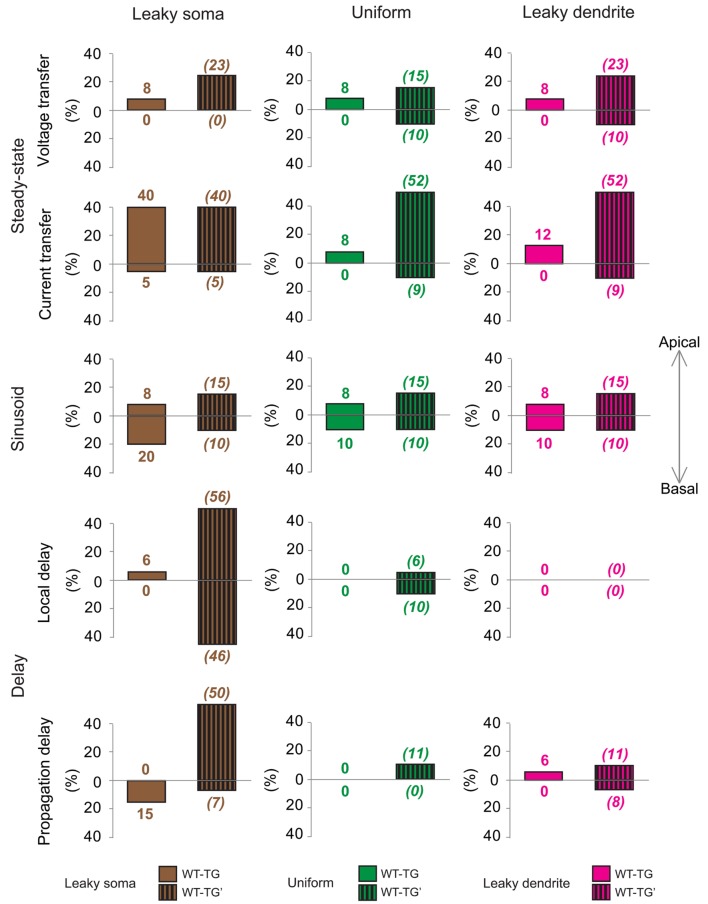
**Effects of morphological degeneration on dendritic signaling is compensated by amyloid-related changes of the plasma membrane in TG neurons.** Percentage differences between TG and WT (solid bars) as well as between TG’ and WT (striped bars) neurons are drawn as percentages of the number of ranges in comparison graphs where statistically significant difference was found in TG-WT and TG’-WT comparisons relative to the total number of ranges where statistical tests were performed. Therefore, 100% difference would mean entirely (significant difference in all ranges of comparison graphs) different dendritic impulse propagation in the compared neurons, while 0% difference means no difference (compared neurons do not differ significantly in any range of comparison graphs). Note that bars with striped lines tend to be bigger than filled bars depicting smaller differences in dendritic signaling between WT and TG neurons with altered membrane than between WT and TG’ neurons with no alteration in membrane properties (no compensation for dendritic atrophy). Brown, green, and magenta colors refer to leaky soma, uniform and leaky dendrites membrane models. Summarized data are based on Supplementary Figures S1–S5.

To quantify the overall efficiency of the presumed compensation process, we considered those ranges in comparison graphs (Supplementary Figures S1–S5) where dendritic signaling significantly differed in TG’ and WT neurons. These ranges represented cases where aberrant dendritic morphology caused significant alterations in dendritic signaling and was hypothesized to be compensated partly or fully by amyloid-driven changes in electrical properties of the neuronal membrane. *Successful compensation* for dendritic atrophy was indicated by the identical PSP transfer or delay properties in WT and TG neurons over the same range where WT and TG’ neurons differed. *Failed compensations* were indicated by persisting significant differences between WT and TG neurons in these ranges. Considering all PSP transfer and delay properties examined, in most of the ranges, Aβ-mediated alterations of the neuronal membrane reduced the size of difference in PSP propagation between WT and TG’ neurons (**Figure 5**, Supplementary Figures S1–S5). Thus compensation occurred, and was successful, since WT and TG neurons had statistically identical PSP propagation properties following compensation. Pathological morphology-caused alterations were the biggest, and therefore compensation by changing neuronal membrane was needed the most, in the *current transfer* of apical dendritic arbors. Considering current transfer in the uniform model, there was a difference between apical current transfers of WT and TG’ neurons in 52% of ranges (**Figure 5**). After compensation, the percentage of ranges where WT and TG neurons differed was only 8%, indicating that in most of the ranges compensation by amyloid-related membrane changes eliminated deviations from normal current transfers, which were caused by morphological degeneration. Dendritic atrophy had the smallest effect on the *local and propagation delays.* Following compensation, no difference was found between local delays of PSPs in WT and TG neurons in uniform and leaky dendrite membrane models. Overall success rates in compensation (number of ranges where compensation was successful divided by the number of ranges where compensation was needed) were 62, 79, and 72% in leaky soma, uniform and leaky dendrite models respectively.

### Synaptic Input Pattern Recognition May Remain Unaltered in Morphologically Transformed TG Neurons Due to Compensatory Changes in Membrane Properties

In the previous section we investigated if signal conduction properties of single PSPs are altered in response to pathological changes in TG neurons, or the effects of morphological alterations may be compensated fully or partially by changes in the neuronal membrane going on simultaneously with dendritic atrophy. However, in reality, neurons usually receive not a single but multiple synaptic inputs generating overlapping PSPs traveling simultaneously along dendrites toward the soma. This synaptic integration results in a summated PSP at the axon hillock, and the size of this summated PSP affects firing activity of neurons, and forms the basis of the ability of neurons to discriminate among different activation patterns of synapses, which are important in learning and memory. To account for this vital process, in the next step, we investigated if this pattern recognition capacity of TG neurons is altered under the effect of high hAPP levels in mutant animals, or pathological effects of morphological alterations on pattern recognition are also compensated by concurrent amyloid-driven alterations of the plasma membrane of TG neurons.

We investigated this problem by using a simple approach based on results of a recent, very detailed study on the correlation between electrotonic properties and synaptic input pattern recognition capabilities of 100s of 1000s different model neurons (de Sousa et al., 2015). In this study it was shown that there is a strong correlation between the pattern recognition capacity of neurons and the mean and variance of electrotonic distances of dendritic synapses from the soma. Therefore, we computed these critical electrotonic parameters of synaptic distributions, as predictors of synaptic activation pattern recognition capacity, in WT, TG, and TG’ neurons (see Materials and Methods) to estimate and compare their pattern recognition performance.

**FIGURE 6:**
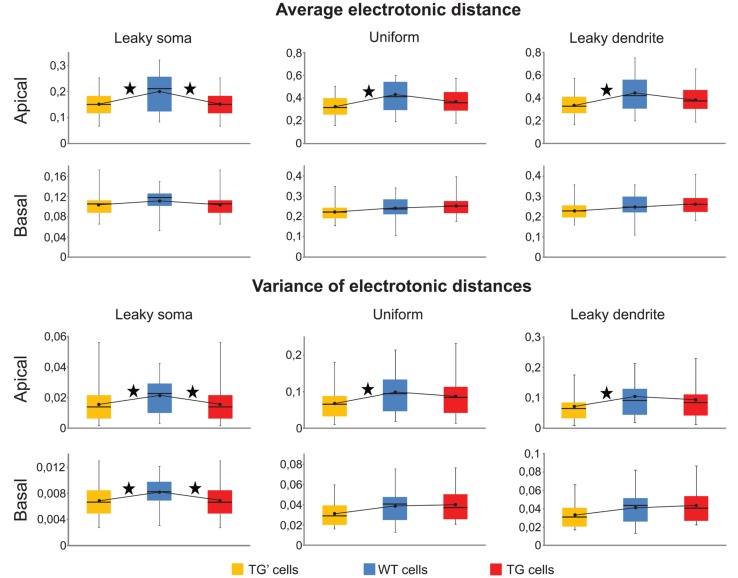
**Synaptic input pattern recognition was preserved in uniform and leaky dendrite models of TG neurons.** Means and variances of electrotonic distances of synapses in apical and basal dendritic arbors of WT (blue), TG (red), and TG’ (yellow) neurons were computed to compare ability of synaptic input pattern recognition in leaky soma, uniform and leaky dendrites models of these neurons. Box plots show the maximum, 75, 50, and 25% percentiles, and the minimum values. Black dots represent the mean values of computed electrotonic parameters within a population of neurons. Asterisks, above lines, interconnecting black dots, mark signifficant differences in these mean values (Mann–Whitney test, *p* < 0.05), and predict dissimilar synaptic input pattern recognition capacity in the respective neuron populations.

Means and the variances of electronic distances of synapses were the same (Mann–Whitney test, *p* > 0.17) both in the apical and in the basal dendritic arbors of the TG and WT neurons in the uniform and leaky dendrite models. Thus, these calculations predicted identical pattern recognition of TG and WT neurons in these models (**Figure 6**). However, apical arbors of TG neurons were predicted to have altered pattern recognition ability based on the significant differences found in both critical parameters (Mann–Whitney test, *p* < 0.03) in TG-WT comparisons with the leaky soma model. Regarding basal dendrites of the neurons in the leaky soma model, our computations were not conclusive since mean electrotonic distances were the same (Mann–Whitney test, *p* > 0.1), while the variances of these distances were statistically different (Mann–Whitney test, *p* < 0.04) in TG and WT neurons.

Since both the identical means and identical variances of electrotonic distances predicted unchanged pattern recognition capabilities in the TG neurons with uniform and leaky dendrite models, it was feasible to ask for the reason behind this preserved pattern recognition. We, again, hypothesized that this was due to the same, amyloid-driven compensatory membrane alterations in TG neurons, which have been shown to be responsible for the surprising similarity of PSP propagation in TG and WT neurons. To check if this compensatory mechanism may be responsible for the conserved recognition ability of synapse activation patterns in TG neurons, pattern recognitions of TG’ neurons were estimated by the two critical parameters and then compared against WT neurons. In uniform and leaky dendrite models, both the means and the variances of electrotonic distances of presumed synaptic sites were different in the apical arbors of TG’ and WT neurons (**Figure 6**, Mann–Whitney test, *p* < 0.03), while no difference was found in these predictors of pattern recognition when the apical arbors of TG and WT neurons were compared (Mann–Whitney test, *p* > 0.27). This analysis, therefore, showed consistently that preservation of healthy level of pattern recognition in apical arbors of TG neurons is due to compensatory changes in membrane conductance and capacitance that occur under the effect of hAPP. These membrane alterations compensated dendritic atrophy in the apical dendrites. At the same time, they did not destroy WT level pattern recognition in basal dendrites of TG neurons where the extent of atrophy is small enough not to alter pattern recognition significantly even without the membrane alterations, as highlighted by the statistically identical critical parameters for basal dendrites in WT-TG’ comparisons. The compensation is successful in neurons with uniform soma-dendrite membrane or with leaky dendrites but it failed to prevent neurons from amyloid-related pathological alterations of pattern recognition if the leaky soma model was used. This may be because only a small fraction of the total soma-dendritic membrane (the membrane of the soma) was allowed to undergo compensatory changes in the leaky soma model, while in the uniform and leaky dendrite models either the entire soma-dendritic membrane or its vast majority (the dendritic part) was allowed to take part in compensation. Alternatively, it is also possible that the leaky soma model approximates distribution of leakage conductance the least faithfully in the soma-dendritic membrane of these neurons.

## Discussion

### Excitability of Transgenic Neurons

The identical thresholds, rising times and amplitudes of single action potentials measured in response to somatic current injections as well as the same firing frequencies elicited by increasing depolarizing current steps in control and Tg2576 transgenic neurons combined with their identical resting membrane potentials (Rocher et al., 2008) suggest similar apparatus for action potential generation in WT and TG neurons. Neuronal excitability is therefore unlikely to be affected significantly by the altered somatic membrane and distorted morphological properties in TG neurons but it may depend on the pattern and strength of afferent activation, and properties of dendritic impulse propagation from the sites of active synaptic contacts to the soma in these neurons. Activation pattern of synapses is unknown but our simulations allow some insight into how excitability in TG neurons may be shaped.

We observed a general tendency of conservation in the rates of current and voltage transfers (except for the current transfer in apical dendrites in the leaky soma model) and in delays of propagating subthreshold PSPs in TG neuron dendrites. Our computations showed that predictors of synaptic input pattern recognition ability are also preserved in TG neurons. Consequently, subthreshold synaptic integration properties should remain very similar in TG neurons. Assuming a spatiotemporally similar pattern of synapse activation over the dendrites in the WT and TG pyramidal neurons, the identical synaptic integration would lead to identical depolarisations, preserving firing probability at identical thresholds on the soma.

However, altered excitability of cortical neurons have been reported based on hypersynchrony and aberrant excitatory neuronal activities seen in AD-related transgenic mice (Palop et al., 2007; Bezzina et al., 2015), and on the frequent seizures (Amatniek et al., 2006; Scarmeas et al., 2009; Vossel et al., 2013; Spencer, 2014) and lowered motoric threshold in AD patients (Di Lazzaro et al., 2003, 2004). The existence of cortical hyperexcitability in AD and in its animal models, despite the loss of excitatory synapses and the nearly identical subthreshold signal transfer and integration properties in TG and WT neurons, restrict the possible origins of such malfunctions in pyramidal neurons. Alterations in neuronal excitability may be caused by the significantly altered type/number/distribution of voltage-dependent channels in TG neurons that may aid action potential generation by amplification of subthreshold PSPs along the dendrites. In addition, the reason for hyperexcitability may be the altered number, strength and pattern of excitatory and inhibitory synaptic inputs the post-synaptic neuron receives from various afferents. The latter possibility seems more likely and dominant because of the unchanged properties of action potential generation in TG neurons of APP mice (Rocher et al., 2008) and because of the many alterations in the post-synaptic receptors and afferent systems in Tg2576 mice (Alpar et al., 2007) and in AD patients (Nordberg, 1992). These and other alterations are likely to act in concert. However, our simulations make it unlikely that alterations in subthreshold dendritic impulse propagation play a major role in shifting TG neurons toward aberrant excitability.

### Significance of Compensation of Morphological Changes by Altered Neuronal Membrane in TG Neurons and in Other Biological Systems

Compensation for changing morphology by modification of passive membrane properties so that major electrotonic properties of the neuron remain unchanged not only preserves relative efficacy of synaptic inputs at any positions in a dendritic tree but also conserves properties of subthreshold synaptic integration. Such conservation of electrotonic structure may be essential for a neuron, and also for the network it is embedded, to maintain its normal function, especially in such a hierarchical stratified circuitry like the cortex (Bekkers and Stevens, 1990). The conservation of subthreshold integration properties of PSPs in very different membrane models of morphologically realistic WT and TG neurons strongly suggests an attempt of the nervous system to counteract the effects of degenerating neuronal morphologies on dendritic impulse propagation and to try to maintain input–output properties at the neuronal and network levels important in normal cortical function. Similar conservations of electric behavior by changing membrane properties to compensate for the altered morphology have also been described in a uniquely identifiable homologous neuron type across species (Cuntz et al., 2013) and in precerebellar hindbrain neurons in the goldfish (Weaver and Wearne, 2008). A very recent publication described that dendritic atrophy itself may also have compensatory effect. Dentate granule cells with dendritic atrophy receive less synaptic contacts and this loss in synapses may be compensated by the increased input resistance of the granule cell, which leads to a precise adjustment in excitability and input-output properties are preserved (Platschek et al., 2016). On the other hand, pathological morphological changes in CA1 pyramidal neurons of the APP/PS1 mouse model of AD lead to more effective integration of PSPs and hyperexcitability (Siskova et al., 2014).

Conservation of subthreshold PSP integration may be especially important in layer II/III neocortical pyramidal neurons known to fire with much lower frequency than nearby GABAegic neurons and excitatory neurons in layers IV and V (Sakata and Harris, 2009; Gentet et al., 2010). Furthermore, layer II/III excitatory neurons in awake mice produce large amplitude (∼20 mV) subthreshold membrane depolarizations driven by synaptic inputs, without leading to action potentials (Petersen et al., 2003; Crochet and Petersen, 2006; Poulet and Petersen, 2008; Crochet et al., 2011). The observed sparse firing activity combined with prominent depolarizations without leading to action potentials in layer II/III neocortical neurons suggest high significance of subthreshold dendritic impulse propagation and synaptic integration in these pyramidal neurons.

Certain morphological types of neuronal alterations have also been conceptualized as compensatory-like responses by the nervous system to try to counteract functional impairments in AD and in other neurodegenerative disorders. These compensatory morphological alterations include size increase of the remaining spines (Fiala et al., 2002), shifts in spine shapes (Dickstein et al., 2010), and size and topological changes of dendrites (Graveland et al., 1985; Arendt et al., 1986, 1995a,b; Scott et al., 1992).

However, it has been shown theoretically that only one type of changes in neuronal dimensions can preserve electrotonic structure and maintain the balance in effectiveness between proximal and distal synaptic inputs without necessary modifications in membrane properties. This is the “uniform isoelectrotonic” mode of morphological changes when dendritic diameters increase (decrease) in proportion with the square of the increase (decrease) in dendritic lengths (Hochner and Spira, 1987; Bekkers and Stevens, 1990; Hill et al., 1994). Layer II/III pyramidal cell dendrites do not follow this rule during dendritic atrophy in AD-related Tg2576 mice, so preservation of WT electrotonic structure is only possible by appropriately scaled alterations in membrane properties of TG neurons. We observed such consequent trends in amyloid-related alterations of specific membrane resistance and capacitance of TG neurons when these membrane properties were fitted to physiological TG neuron resistance and time constant values. Changing leakage resistance and capacitance values in TG neurons resulted in significant shifts in descriptors of dendritic impulse propagation toward the normal values.

Additionally, active membrane properties may also be involved in regulation of signal propagation and synaptic integration in TG and WT neurons. Active dendritic processes have been widely reported in neocortical pyramidal neurons, though these experiments were carried out mainly on layer V pyramidal neurons because they possess thicker and therefore technically more accessible dendrites than layer II/III neurons. However, active backpropagation of action potentials have also been reported in apical dendrites of layer II/III pyramidal neurons supported by voltage-dependent sodium channels (Waters et al., 2003; Waters and Helmchen, 2006) and accompanied by influx of calcium ions (Svoboda et al., 1999; Larkum et al., 2007). On the other hand, the prediction of our simulations is that Aβ-driven appropriate scaling of passive membrane properties may prevent atrophy-caused pathological impairments in dendritic attenuations and delays of subthreshold PSPs, and thus can maintain properties of normal synaptic integration. This finding may strengthen the idea on the special importance of subthreshold dendritic signaling in layer II/III pyramidal neurons, based on their sparse firing activity and large subthreshold membrane potential fluctuations observed experimentally (Sakata and Harris, 2009; Gentet et al., 2010; Crochet et al., 2011).

### Potential Clinical Relevance of Findings

Our findings on unaltered propagation and integration of subthreshold dendritic signals in layer II/III callosal somatosensory TG neurons, combined with the observed similarity in action potential generation properties, as well as the presumed special importance of subthreshold information processing in these neurons, suggest that AD-related changes in excitability of these callosal neurons are primarily due to the pathological number, type, balance, and activation pattern of synaptic *connections* received by the neurons, *rather than information processing failures within neurons* of these networks. Pathological reconfiguration of neurotransmitter systems in vulnerable neural networks have been widely recognized in hAPP mice (Alpar et al., 2007) and in AD (Nordberg, 1992; Di Lazzaro et al., 2003; Ferreri et al., 2003). Our computer models suggest that these alterations in functional connections are not necessarily accompanied by altered signal propagation and processing during amyloid stress. If this is the case in certain stages of AD, then our results may well assist in choosing targets of possible future therapies for these stages of AD: targeting disrupted neurotransmitter systems (failed connectivity) of pathological networks in AD, rather than trying to modulate membrane properties that shape intraneuronal signal processing and excitability might be more promising. In line with this, all currently certified compounds for AD management target either the altered glutamatergic or cholinergic neurotransmitter systems (Frost et al., 2015).

### Limitations of Findings

Our model predictions on unaltered pattern recognition and summation of PSPs were based on neurons of 11 months-old Tg2576 mice, where plaque deposition is submaximal. At later stages, plaque deposition has been shown to be increasing together with the amount of soluble Aβ (Kawarabayashi et al., 2001) in these animals. Further, the extent of morphological degeneration of neuronal dendrites in Tg2576 mice has been proved to be age-dependent (Luebke et al., 2010). Therefore, subthreshold dendritic signaling in TG neurons might also be age-dependent, unless continuous Aβ-mediated changes in dendritic morphology are accompanied by appropriate rescaling of membrane properties, as what happened at the age we studied.

Age-dependent synaptic integration has been examined experimentally in neocortical neurons of Tg2576 mice *in vivo*. It was shown that, in response to transcallosal stimuli, neurons of 14 month-old Tg2576 mice, with significant plaque deposition in the brain, had 2.5-fold greater rate of response failure and twofold reduction in response precision in their evoked synaptic potentials in comparison to neurons of age matched non-transgenic control. This was in contrast with the identical properties of evoked responses found in neurons of 8–10 months-old control and transgenic mice, where plaque deposition has not started (Stern et al., 2004). Spontaneous membrane potential dynamics were unaltered in transgenic neurons at both ages, suggesting unaltered level of overall synaptic innervations in transgenic and control neurons. Based on these findings it was concluded that plaque deposition disrupts successful propagation and integration of information in neurons of 14 months-old transgenic mice. However, these experiments on slightly younger, 8–10 months-old animals support our predictions on unaltered dendritic signaling in 11 months-old transgenic animals, where plaque deposition is still minimal. These age-dependent variances emphasize the possibility that transgenic animal neurons propagate and integrate information differently as amyloid burden increases.

Our findings may not only dependent on age but may also be varying according to neuron types and area of the brain. By using the reconstructed morphology of a healthy CA1 pyramidal neuron equipped with experimentally derived active membrane properties, a model study (Culmone and Migliore, 2012) found that effects of Aβ on intrinsic membrane properties may lead to significant alterations of the activity patterns of the neuron. Based on these findings, the study suggested possible pharmacological interventions to modify channels’ kinetic and activation properties that might counteract amyloidogenic effects in order to restore normal neuronal excitability and activity patterns in AD. However, possible effects of Aβ-related morphological alterations on dendritic signaling of the neuron were not taken into account in this study.

## Conclusion

**FIGURE 7:**
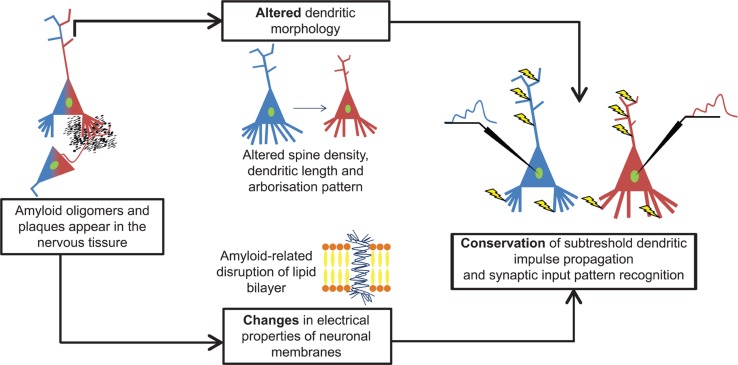
**Amyloid-β alters both dendritic morphology and neuronal membranes in cortical neurons and they compensate each other’s effects on subthreshold dendritic signaling.** Both types of alterations alone are potential sources of pathological changes in dendritic signaling. However, these disturbances act parallel and their combined effect is a kind of compensation that preserves features of subthreshold dendritic signaling and synaptic integration in layer II/III somatosensory pyramidal neurons of 11 months-old hAPP mutant mice. Blue and red colors refer to WT and TG neurons and their signaling properties in response to identical synaptic activation pattern (yellow arrows).

Our computer simulations showed that amyloid-related morphological degeneration and plasma membrane alterations tend to compensate each other’s pathological effects on subthreshold dendritic signal propagation and integration in layer II/III commissural neurons of AD-related 11 months-old transgenic mice (**Figure 7**). This preservation tendency in dendritic signaling is a robust phenomenon, since it has been found in models covering a wide range of soma-dendritic membrane conductance distributions with variable spatial extents of amyloid-driven membrane alterations, and it has also been observed over neuron size-dependent and size-independent, normalized scales. The phenomenon of compensation of dendritic atrophy by amyloid-driven membrane alterations may have important implications for choosing suitable targets of future therapeutic strategies for AD.

## Author Contributions

AS performed most of the computer simulations, analyzed data, created figures, and contributed to revising the paper. ZK contributed to computer simulations, data analysis, statistical tests, and figures. AA reconstructed the neurons, discussed and interpreted the results and contributed to revising the paper. EW designed and organized the study, discussed and interpreted results and wrote the paper.

## Conflict of Interest Statement

The authors declare that the research was conducted in the absence of any commercial or financial relationships that could be construed as a potential conflict of interest.
