# Supplementary material for: A Novel Form of Compensation in the Tg2576 Amyloid Mouse Model of Alzheimer’s Disease
Source: Front Cell Neurosci. 2016 Jun 16;10:152. doi: 10.3389/fncel.2016.00152 (PMC4909742; doi:10.3389/fncel.2016.00152)
Supplement: Supplementary file 1 [file Data_Sheet_1.pdf]

Supplementary Material

A novel form of compensation in the Tg2576 amyloid mouse model of Alzheimer disease

Attila Somogyi, Zoltán Katonai, Alán Alpár, Ervin Wolf*

***Correspondence:** Ervin Wolf
Email: wolf.ervin@anat.med.unideb.hu

Supplementary Results

1. Detailed comparison of subthreshold dendritic impulse propagation in TG, WT and hypothetical TG' neurons

Comparison graphs (Figure S1-S5) are presented. These figures of the Supplementary Material compare different properties of subthreshold dendritic impulse propagation in wild-type (WT), Tg2576 transgenic (TG), and hypothetical TG' neocortical pyramidal neurons of mice by using non-normalized, absolute scales. Data presented in Figure S1-S5 was included in example and summary Figures 4-6 of the body text.

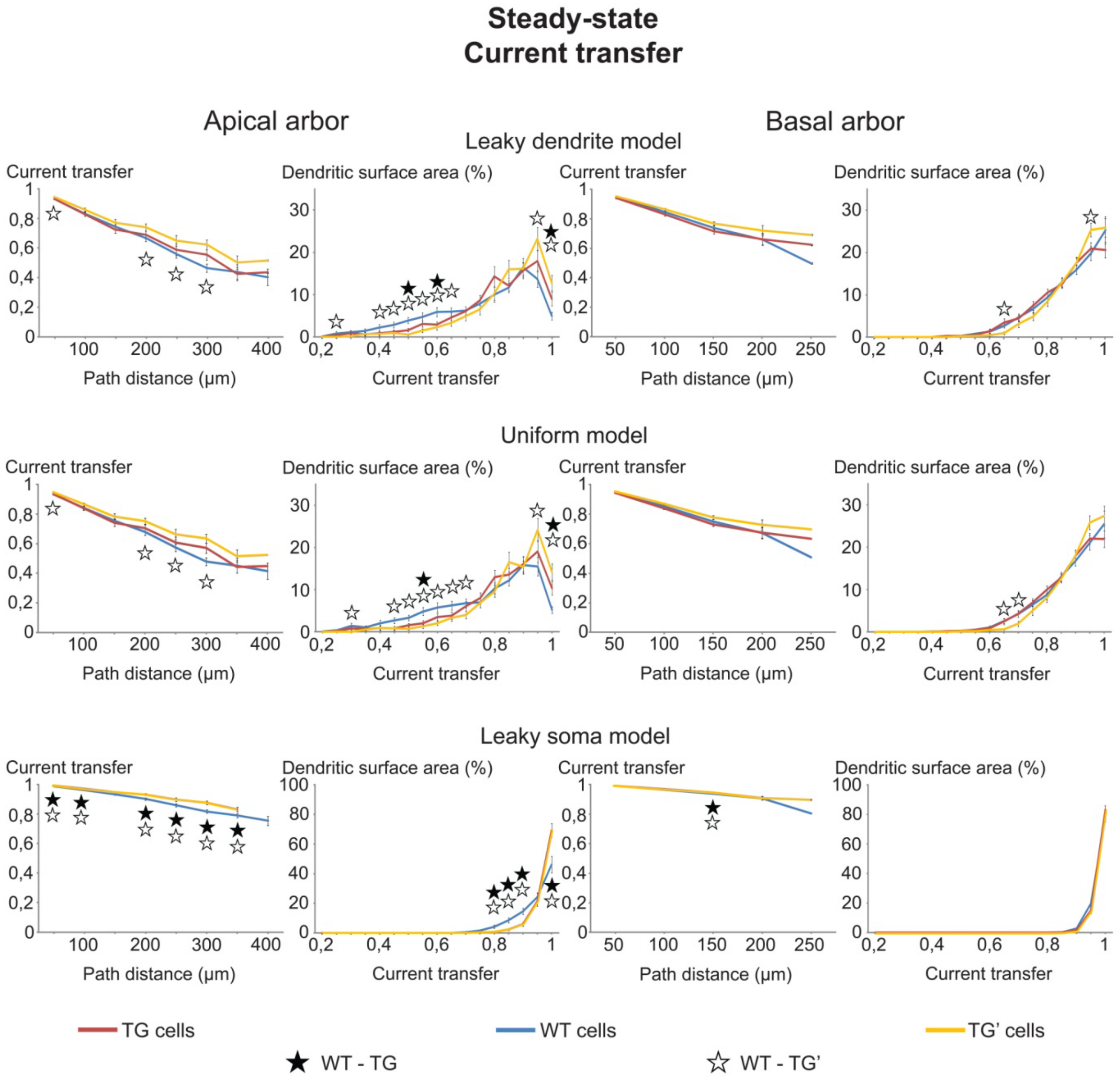

Figure S1 Current transfers of locally generated PSPs in wild-type (WT, blue lines), transgenic (TG, red lines) and hypothetical TG' (yellow lines) neurons measured on absolute scales. Distance dependence of current transfers (first and third column) and percentages of total dendritic surface area (second and fourth column) with different rates of current transfers were examined in three membrane models (see rows). Black (open) asterisks mark path distance and transfer ranges where TG and WT (TG' and WT) neurons were different (Mann-Whitney test, $p<0.05$). TG' neurons represent the hypothetical case when neurons have pathological morphological alterations without the amyloid-driven biophysical changes in the neuronal membrane (see Methods of the body text for details). In ranges with open but without black asterisks, compensation of dendritic atrophy by amyloid –driven membrane alterations was successful. In ranges with both open and closed asterisks compensation failed. In this, and also in the following figures, some lines run very close to one another and some parts of lines remain hidden.

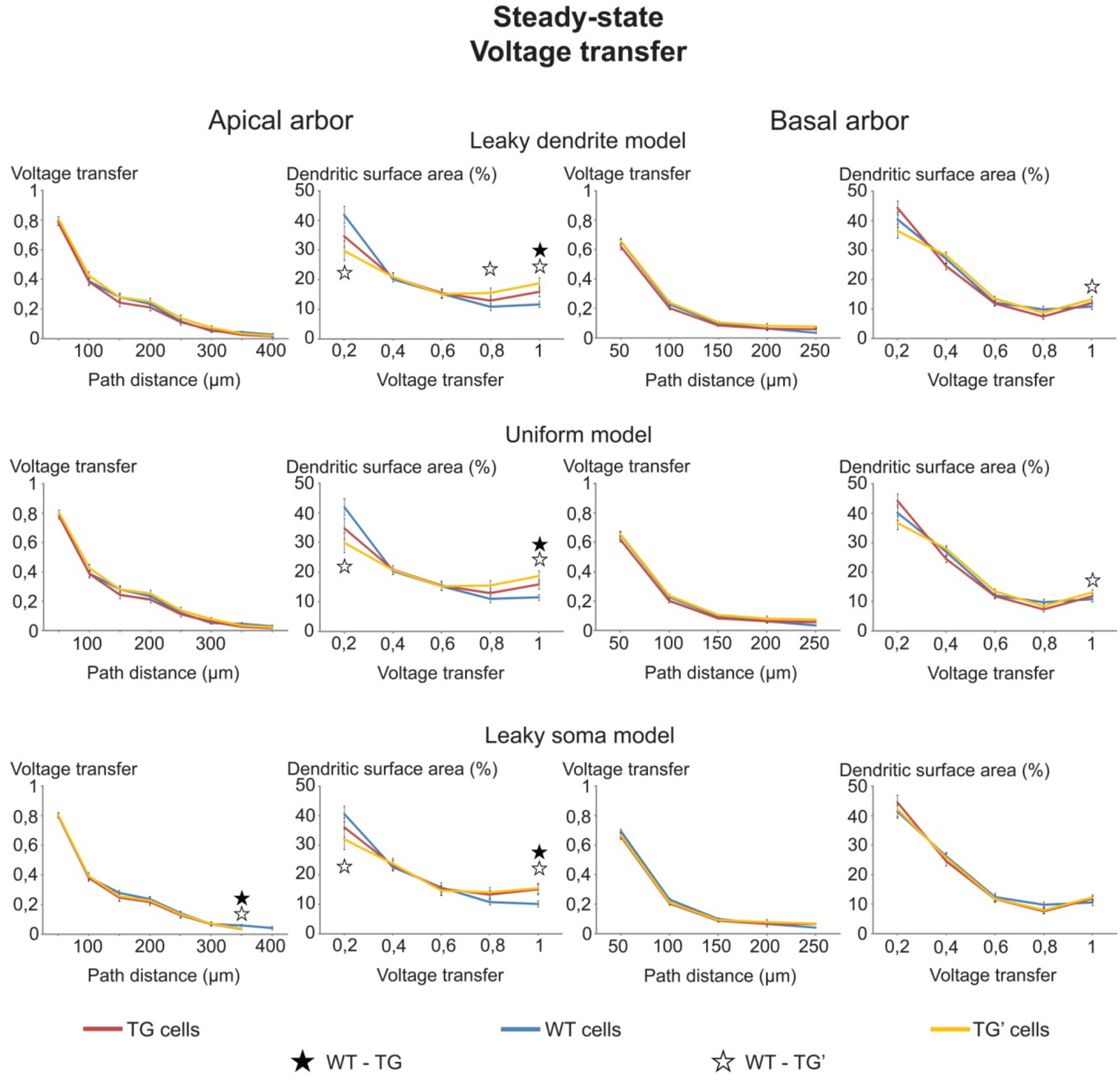

Figure S2 Steady-state voltage transfers (absolute scale)
See legend for analogue Figure S1 for description.

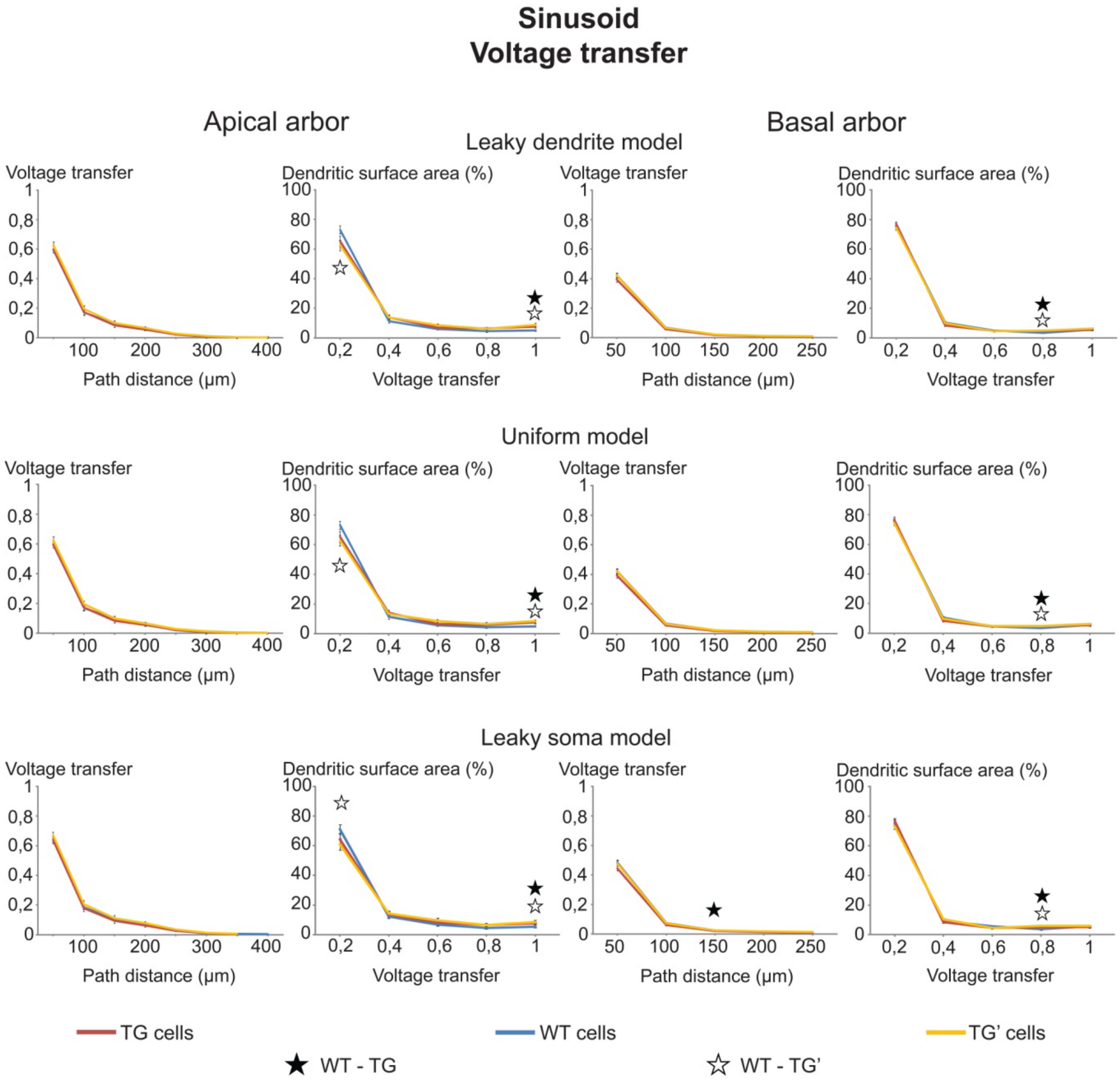

Figure S3 Sinusoid voltage transfer at 50Hz (absolute scale)
See legend for analogue Figure S1 for description.

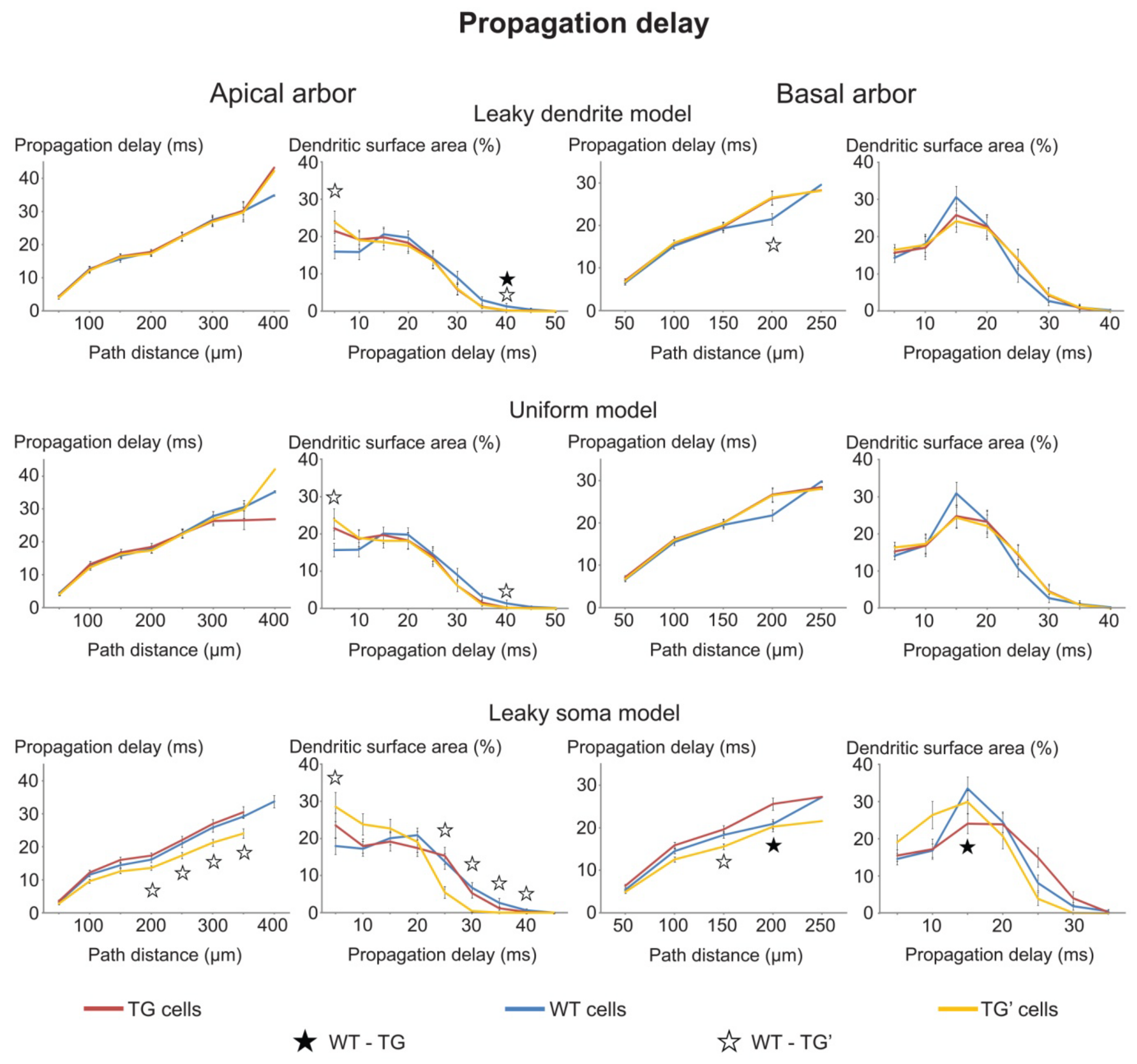

Figure S4 Propagation delays (absolute scale)
See legend for analogue Figure S1 for description.

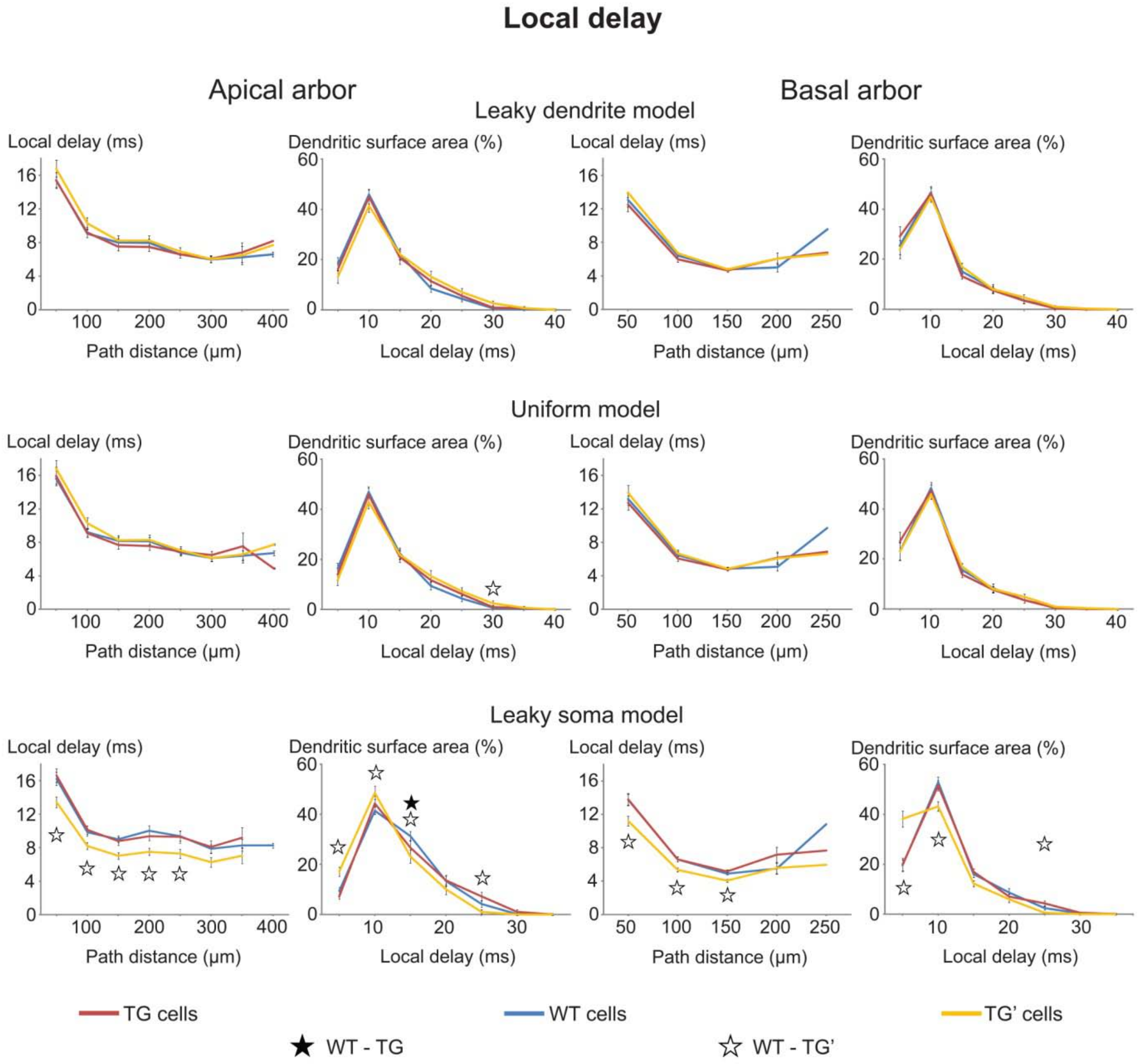

Figure S5 Local delays (absolute scale)
See legend for analogue Figure S1 for description.

2. WT-TG differences in dendritic signaling over normalized scales

Summary figure S6 depicts alterations of dendritic signaling in TG neurons when transfers, delays and path distances were measured over normalized scales, as percentages of their maximum values in neurons to eliminate within-neuron-group variances. This summary figure was based on comparison graphs analogue to those shown in Figures S1-S5, therefore these analogue comparison graphs were not presented in detail.
Generally, WT-TG differences were detected to be small over normalized scales, similarly to those over absolute scales.

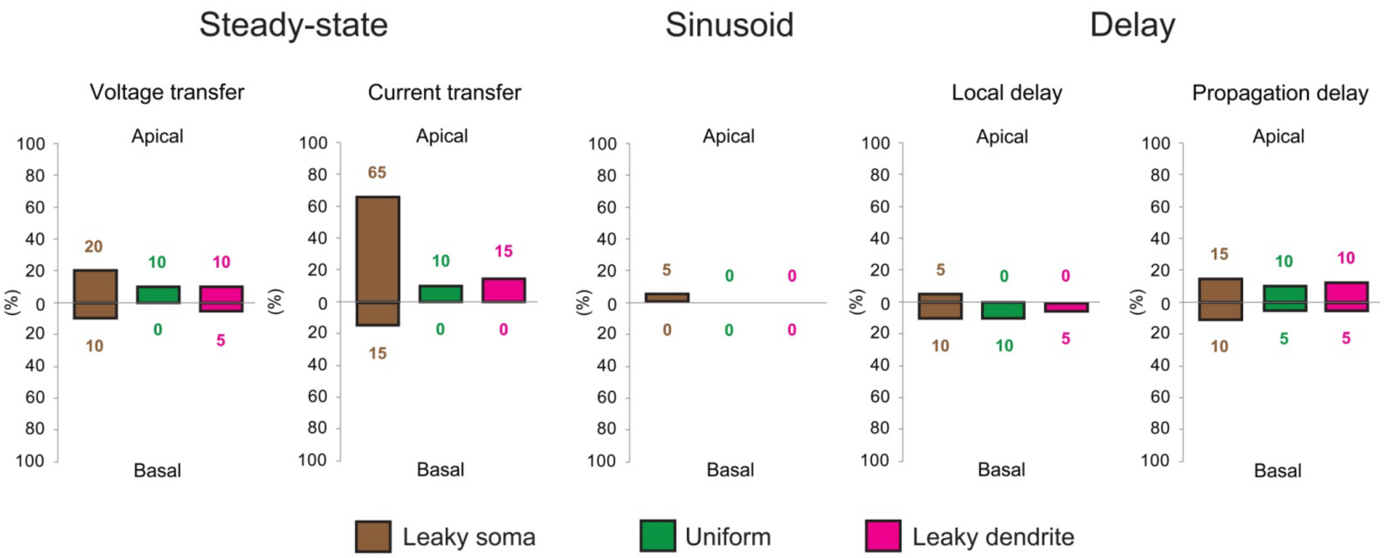

Figure S6 Aβ-driven alterations in steady-state current- and voltage transfers, 50Hz sinusoid voltage transfers and local- and propagation delays of PSPs in TG neurons relative to control, WT neurons (normalized scale). Comparisons of dendritic impulse propagation were summarized in the leaky soma (brown), uniform (green) and leaky dendrite (magenta) membrane models. Heights of bars are proportional to the percentage differences between WT and TG neurons in certain features of dendritic impulse propagation. Upper and lower extensions of a bar visualize the size of differences present in apical arbors of WT and TG neurons and in basal arbors of WT and TG neurons.

3. Compensation over normalized scales

Compensation of morphological atrophy-caused alterations in dendritic signaling by amyloid-driven membrane changes was also examined over normalized scales. These results indicated that compensation by membrane changes is present over normalized scales too, similarly to what was found and described in the body text over absolute scales. Thus, compensation is a phenomenon, which is independent on the absolute or normalized nature of scales used in the analysis.

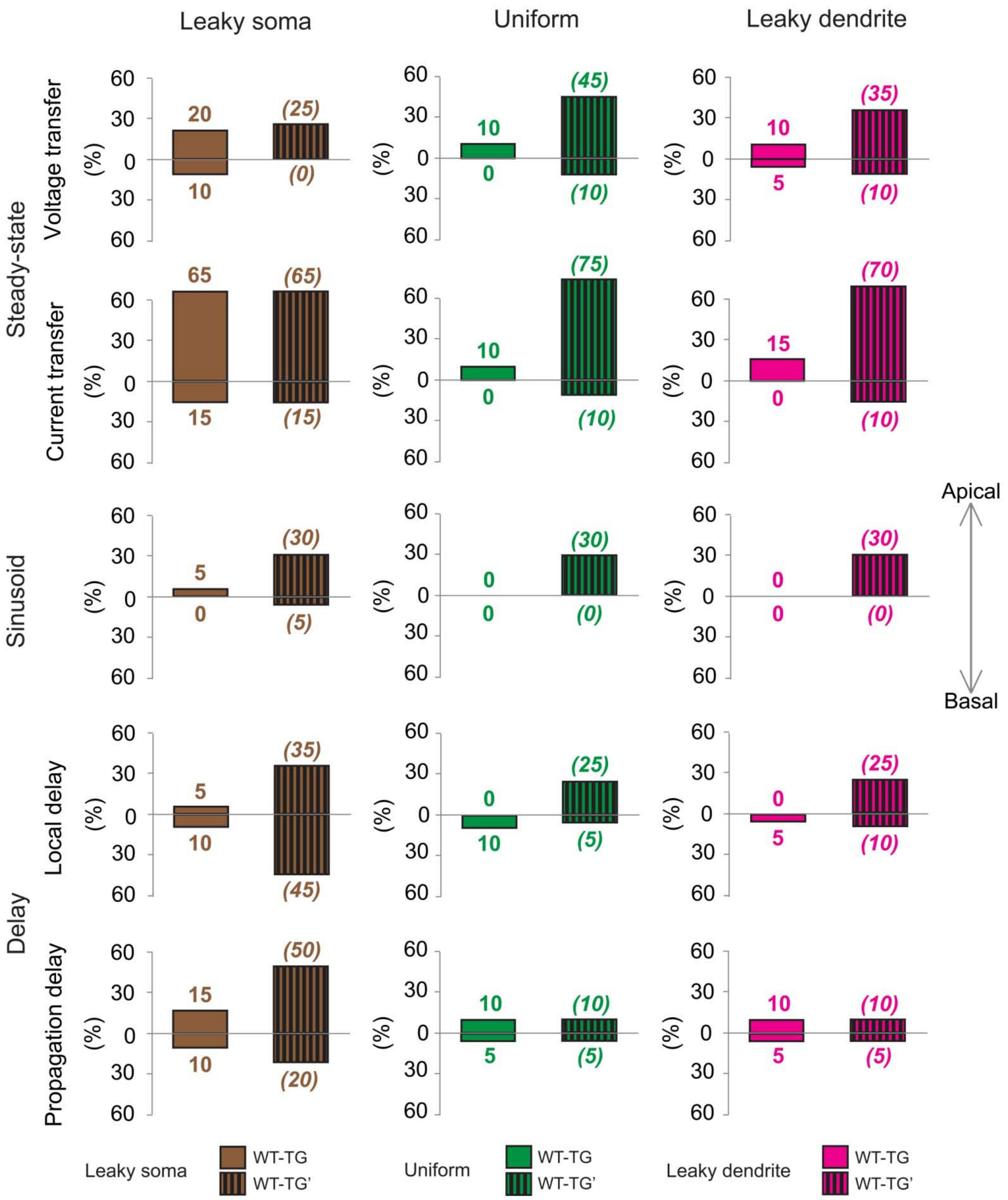

Figure S7 Effects of morphological degeneration on dendritic signaling is compensated by amyloid-related changes of the plasma membrane in TG neurons (normalized scale). Percentage differences between TG and WT (solid bars) as well as between TG' and WT (striped bars) neurons were drawn as percentages of the number of ranges in comparison graphs (not shown), where statistically significant difference was found in TG-WT and TG'-WT comparisons relative to the total number of ranges where statistical tests were performed. Therefore, 100% difference (significant difference in all ranges of comparison graphs) would mean entirely different dendritic impulse propagation in the compared neurons,

while 0% difference means no difference (compared neurons did not differ significantly in any range of comparison graphs). Note that bars with striped lines tend to be bigger than filled bars. This indicates smaller differences in dendritic signaling between WT and TG neurons than between WT and TG' neurons. Brown, green and magenta colors refer to leaky soma, uniform and leaky dendrites membrane models.

4. The other side of the coin: Would neurons perform worse than TG neurons if amyloidosis did not cause morphological atrophy, rather affected only their neuronal membrane properties?

Dendritic signaling is dependent on both morphology and membrane properties of dendrites and their deviations from the normal (WT) case may affect signaling. However, if both morphology and neuronal membrane get altered simultaneously, as it happens during amyloidosis, signaling may remain virtually unchanged, as we showed. Since morphological atrophy is more easily and reliably detectable than membrane alterations in TG neurons, we started investigating the compensation phenomenon by creating hypothetical TG' neurons with well-defined pathological morphology but with, unaltered, healthy membrane properties. In relation to TG' neurons, we asked if TG' neurons (with only morphological alterations) deviate more than TG (with both morphological and membrane alterations) neurons from WT neurons? However, it is also possible to ask if neurons with healthy morphology but with amyloid-driven altered membrane properties (WT' neurons) deviate more than TG neurons from WT neurons? We created such hypothetical WT' neurons by assuming homogeneous soma-dendritic membrane. Then, we compared signaling of these WT' and normal WT neurons by using methods analogue to those used for TG'-WT-comparisons. We found that differences in subthreshold dendritic signaling between WT' and WT neurons are bigger than the differences between TG and WT neurons. This finding emphasized again: Normal dendritic signaling is preserved by concurrent, amyloid-driven alterations of morphology and membrane properties of dendrites in TG neurons. However, if the two kinds of alterations do not compensate each other's effects, signaling in neurons becomes more altered than during concurrent alterations of these two properties (Figure S8). The two kinds of alterations are scaled suitably under the effect of amyloid, resulting in conservation of subthreshold dendritic signaling.

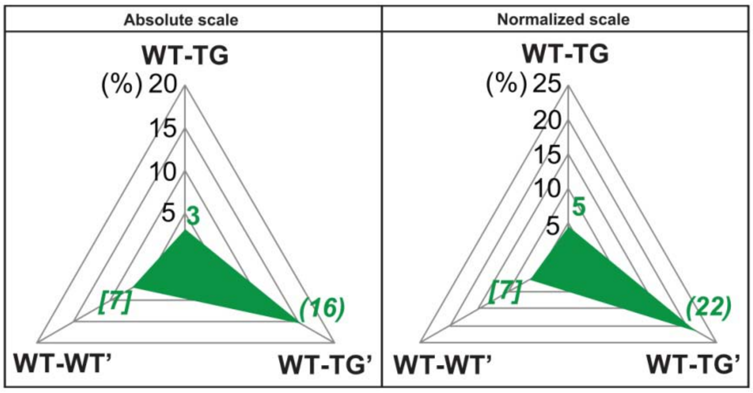

Figure S8 Combined and individual effects of amyloid-driven membrane and morphological alterations on subthreshold dendritic signaling. Extensions of polygons along WT-TG, WT- TG' and WT- WT' axes measure percentage alterations in subthreshold dendritic signaling from the normal (WT) case in AD-related transgenic (TG) neurons (with both morphological and membrane alterations), and in hypothetical TG' and WT' neurons with only one type of amyloid-driven alteration. Alteration in dendritic signaling is the smallest in TG neurons, where amyloidosis led to alterations in both morphology and in membrane properties, i.e., compensation occurred.

Supplementary Discussion

Efficiency of compensation in different soma-dendritic membrane models

By comparing the three membrane models, the extent and number of alterations in dendritic impulse propagation of TG neurons were the biggest in the leaky soma model and the smallest in the uniform model. This may, at least in part, be explained by an important feature of these models of conductance distributions over the soma-dendritic membrane. The soma represented only a few percentage of the total soma-dendritic membrane surface area of these neurons, and membrane resistance was varied only on this tiny part of the neuronal membrane during fitting neuronal resistance to altered TG morphologies in the leaky soma model, while dendritic membrane resistance was kept constant (~assumed to be less affected by amyloidal burden). On the other end of the spectrum of our membrane models, the entire soma-dendritic membrane was assumed to be affected by Aβ and resistance of the entire soma-dendritic membrane of TG neurons was varied during the fitting procedure in case of uniform membrane model. The leaky dendrite model is in the middle among the three membrane models in this respect, where most of the soma-dendritic membrane (the dendritic part) was assumed to be affected by Aβ in the TG neurons and only the smaller, somatic membrane was considered as unaltered (~assumed to be altered to a smaller degree relative to dendritic alterations). Thus, as bigger and bigger part of the soma-dendritic membrane was assumed to be involved in the toxic effect of Aβ in the different membrane models (soma only, dendrites only, soma and dendrites), and included in fitting of specific membrane resistance to electrophysiological neuron resistance, the degree of differences in dendritic impulse propagation between the TG and WT neurons was getting less and less due to the improving compensation by altered membrane properties. Experimentally, Aβ-mediated alterations of the neuronal membrane have been reported in all parts of the neuronal membrane (Alpar et al., 2006;Garcia-Marin et al., 2009;Leon-Espinosa et al., 2012;Deng et al., 2014). However, it is still not clear whether or not the degree of Aβ-mediated alterations of the plasma membrane is the same in the somatic and dendritic compartments.

Regarding another aspect of our membrane models, the distribution of leakage conductances in the membrane of layer II/III somatosensory pyramidal neurons has not been studied in wild-type and Tg2576 mice in a comparative manner. However, wild-type pyramidal neurons in CA1 layer of hippocampus and also in neocortical layer V have been reported to have non-uniform specific membrane resistances over their soma-dendritic membrane and the resistance was smaller in the dendritic than in the somatic compartment in most of these neurons (Stuart and Spruston, 1998;Golding et al., 2005). Extrapolating these findings, we may speculate that our leaky dendrite membrane model, with the $R_{md}<R_{ms}$ assumption, might be the most faithful approximation for the distribution of membrane conductances in our somatosensory pyramidal neurons. It is noteworthy that compensation for dendritic atrophy by altered neuronal membrane was more efficient and differences between TG and WT neurons were smaller in our leaky dendrite model than in the leaky soma model with the opposite, $R_{ms}<R_{md}$ assumption.

Supplementary references

Alpar, A., Ueberham, U., Bruckner, M.K., Seeger, G., Arendt, T., and Gartner, U. (2006). Different dendrite and dendritic spine alterations in basal and apical arbors in mutant human amyloid precursor protein transgenic mice. *Brain Research* 1099**,** 189-198. doi: DOI 10.1016/j.brainres.2006.04.109.

Deng, X., Li, M., Ai, W., He, L., Lu, D., Patrylo, P.R., Cai, H., Luo, X., Li, Z., and Yan, X. (2014). Lipolysaccharide-Induced Neuroinflammation Is Associated with Alzheimer-Like Amyloidogenic Axonal Pathology and Dendritic Degeneration in Rats. *Adv Alzheimer Dis* 3**,** 78-93. doi: 10.4236/aad.2014.32009.

Garcia-Marin, V., Blazquez-Llorca, L., Rodriguez, J.R., Boluda, S., Muntane, G., Ferrer, I., and DeFelipe, J. (2009). Diminished perisomatic GABAergic terminals on cortical neurons adjacent to amyloid plaques. *Frontiers in Neuroanatomy* 3. doi: Artn 28

Doi 10.3389/Neuro.05.028.2009.

Golding, N.L., Mickus, T.J., Katz, Y., Kath, W.L., and Spruston, N. (2005). Factors mediating powerful voltage attenuation along CA1 pyramidal neuron dendrites. *Journal of Physiology-London* 568**,** 69-82. doi: 10.1103/jphsiol.2005.086793.

Leon-Espinosa, G., DeFelipe, J., and Munoz, A. (2012). Effects of Amyloid-beta Plaque Proximity on the Axon Initial Segment of Pyramidal Cells. *Journal of Alzheimers Disease* 29**,** 841-852. doi: Doi 10.3233/Jad-2012-112036.

Stuart, G., and Spruston, N. (1998). Determinants of voltage attenuation in neocortical pyramidal neuron dendrites. *Journal of Neuroscience* 18**,** 3501-3510.
